# Resting right ventricular function in pectus excavatum: do haller index and age matter?

**DOI:** 10.3389/fsurg.2025.1685170

**Published:** 2025-11-06

**Authors:** Jungsuk Choi, Gongmin Rim, Seha Ahn, June Lee, Jin Yong Jeong

**Affiliations:** 1 Department of Thoracic and Cardiovascular Surgery, The Catholic University of Korea, Eunpyeong St. Mary’s Hospital, Seoul, Republic of Korea; 2 Department of Thoracic and Cardiovascular Surgery, CHA University Bundang CHA Hospital, Seongnam, Republic of Korea; 3 Department of Thoracic and Cardiovascular Surgery, Uijeongbu Eulji Medical Center, Eulji University, Uijeongbu-si, Republic of Korea; 4 Department of Thoracic and Cardiovascular Surgery, Incheon St. Mary’s Hospital, College of Medicine, The Catholic University of Korea, Seoul, Republic of Korea

**Keywords:** pectus excavatum, right ventricular function, haller index, transthoracic echocardiography, TAPSE, fractional area change

## Abstract

**Background:**

Pectus excavatum (PE) is associated with compression of intrathoracic structures, potentially impairing right ventricular (RV) function. The Haller index (HI) is widely used to quantify the severity of deformities; however, its functional correlation remains uncertain. This study aimed to evaluate whether HI and patient age were associated with resting RV systolic function in patients with PE.

**Methods:**

This retrospective cohort study analyzed 67 patients with PE who underwent surgical correction and had complete preoperative echocardiographic data between January 2013 and January 2024. RV function was assessed using the fractional area change (FAC) and tricuspid annular plane systolic excursion (TAPSE). Patients were stratified by HI severity (<3.2, 3.2–3.4, ≥3.5) and age (≤13 vs. >13 years). Receiver operating characteristic (ROC) analysis and multivariate regression were used to determine predictors of RV dysfunction.

**Results:**

The mean HI was 4.09 (±0.99), and the mean age was 15.85 (±4.96) years. Neither HI nor age significantly correlated with FAC (HI: *r* = –0.04, *p* = 0.749; age: *r* = 0.09, *p* = 0.455) or TAPSE (HI: *r* = –0.05, *p* = 0.685; age: *r* = 0.14, *p* = 0.245). Subgroup and regression analyses confirmed that neither HI nor age were predictive of impaired RV systolic function. ROC analysis demonstrated the poor discriminatory power of age for reduced FAC (area under the curve = 0.478).

**Conclusion:**

Resting RV systolic function was not significantly influenced by HI or age in patients with PE. These findings highlight the need for a functional, individualized approach beyond anatomical metrics for surgical evaluation.

## Introduction

Pectus excavatum (PE) is the most common congenital chest wall deformity and is characterized by posterior displacement of the sternum and adjacent costal cartilage. It affects approximately 1 in 400 live births and has a strong male predominance (1, 2). Although historically regarded as a cosmetic issue, accumulating evidence indicates that PE can result in physiological compromise, particularly involving cardiopulmonary function (3–5). Patients often report exertional dyspnea, fatigue, reduced exercise tolerance, and palpitations, many of which improve after surgical correction (6, 7). The anatomical severity of PE is commonly assessed using the Haller index (HI), which is calculated as the ratio of the transverse thoracic diameter to the anteroposterior distance at the point of maximal sternal depression on computed tomography (CT). An HI > 3.25 is frequently cited as an indication for surgical intervention (8, 9). However, the relationship between HI and functional impairment remains unclear. Some studies suggested that high HI values are associated with cardiac compression and reduced ventricular filling, whereas others found preserved cardiac function even in cases of severe anatomical deformities (10–12). Given its anterior location and the thin myocardial wall, the right ventricle (RV) may be particularly susceptible to extrinsic compression during PE. Echocardiographic measures such as fractional area change (FAC) and tricuspid annular plane systolic excursion (TAPSE) are recommended indicators of RV systolic performance and widely used in pediatric and young adult populations because of their reproducibility and ease of acquisition (13). However, few studies have examined whether HI correlates with specific RV functional indices (14, 15). In addition to anatomical severity, age may influence the physiological effects of PE. It has been hypothesized that prolonged mechanical compression during growth could contribute to RV remodeling and dysfunction over time (16). However, data on age-related differences in RV function, particularly within the defined HI strata, are limited and inconclusive. Therefore, the objectives of this study were: (1) to evaluate the association between PE anatomical severity, as measured using HI, and resting RV systolic function assessed by echocardiography, and (2) to determine whether age influences RV function across HI severity categories.

## Materials and methods

### Study design and population

**Figure 1:**
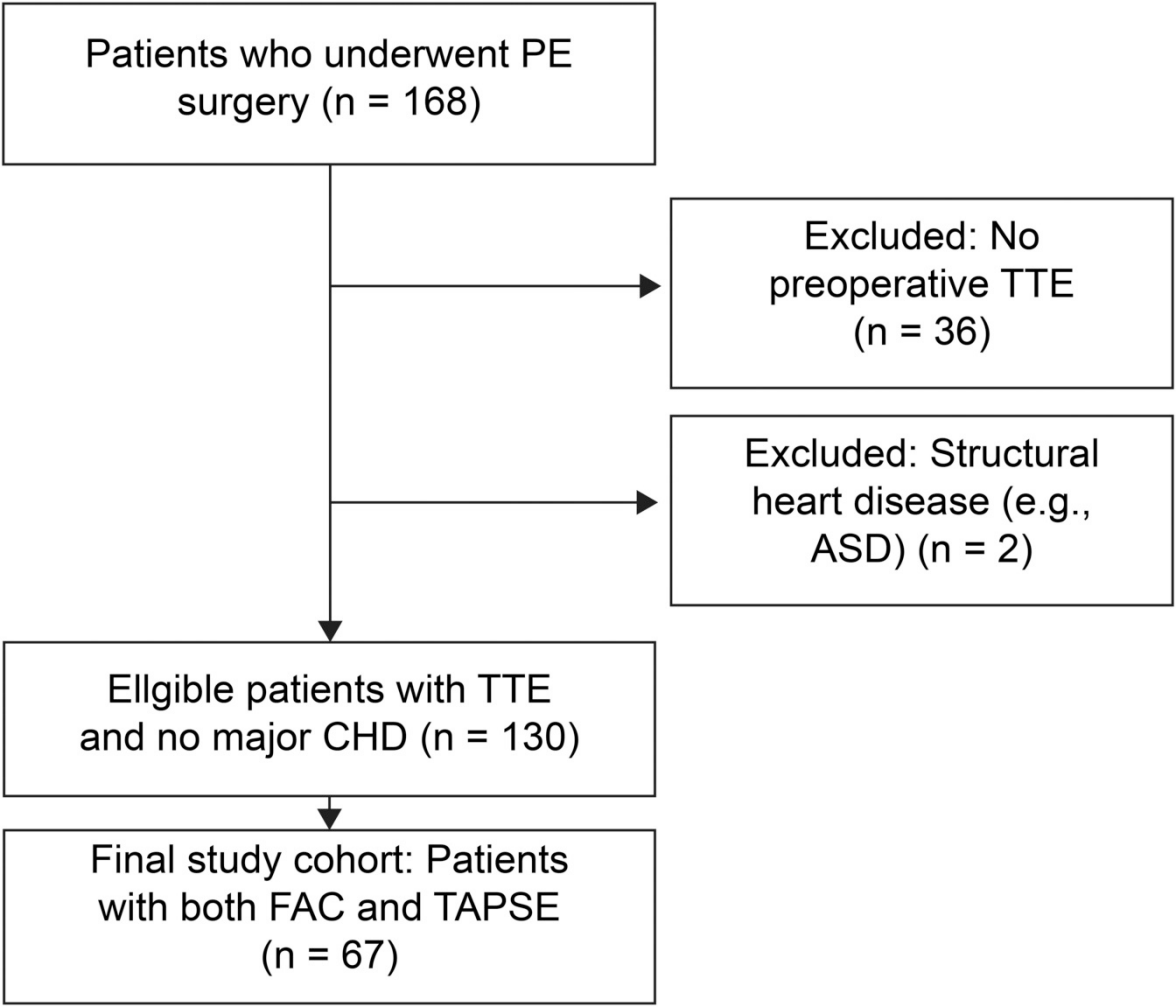
Patient selection flowchart. PE, pectus excavatum; TTE, transthoracic echocardiography; FAC, fractional area change; TAPSE, tricuspid annular plane systolic excursion.

This retrospective cohort study was conducted at a single tertiary center and approved by the Institutional Review Board (IRB No. OC25RISI0129). The requirement for written informed consent was waived by the IRB owing to the retrospective design and use of de-identified data. The study was conducted in accordance with the Declaration of Helsinki. We screened 168 patients who underwent surgical correction of PE between January 2013 and January 2024. The inclusion criterion required complete preoperative transthoracic echocardiography (TTE) data, including FAC and TAPSE measurements. Patients with prior cardiothoracic surgery, congenital heart disease, or incomplete imaging data were excluded. After applying these criteria, 67 patients were included in the final analyses (Figure 1). Demographic and clinical information, including age, HI, and RV function parameters, were extracted from electronic medical records. All the surgical procedures were performed by the corresponding author (J.J.Y.). Quantitative assessments were performed using preoperative CT and echocardiography. For subgroup analysis, HI was stratified into three categories: mild (<3.2), moderate (3.2–3.4), and severe (≥3.5). Although an HI > 3.25 is traditionally regarded as a surgical threshold (8), a cutoff of 3.5 has been adopted in prior studies to delineate patients with more severe anatomical distortion and functional symptoms (14, 15). This stratification enabled a more nuanced assessment of potential dose–response relationships between HI severity and RV function while preserving statistical balance across groups. Age-based grouping was based on receiver operating characteristic (ROC) curve analysis, which identified 13 years as the optimal cutoff for predicting reduced RV systolic function. Although data-driven, this threshold also approximates the age of pubertal transition and thoracic growth acceleration, which may affect the structural impact of PE. Accordingly, patients were classified as younger (≤13 years) or older (>13 years) for subgroup comparisons.

### Chest computed tomography and haller index

Preoperative and postoperative chest CT scans were obtained to evaluate thoracic anatomy and volumetric changes. Preoperative CT imaging was performed as part of the standard presurgical workup to establish baseline anatomical and morphological parameters. All CT scans were acquired using a multi-detector CT system with a 1-mm slice thickness to ensure high-resolution images suitable for precise three-dimensional reconstruction and volumetric analysis. A noncontrast protocol was employed in all cases to avoid the potential risks associated with intravenous contrast agents, particularly in the predominantly pediatric patient population. HI, a standard metric for quantifying the severity of PE, was calculated from axial CT images. It was defined as the ratio of the transverse diameter of the thoracic cavity to the shortest anteroposterior distance between the posterior surface of the sternum and anterior aspect of the vertebral body at the point of maximal sternal depression.

### Echocardiographic assessment of RV function

Preoperative TTE was performed using a commercially available ultrasound system (Philips EPIQ CVx, GE Vivid E95, Siemens ACUSON SC2000). Resting RV systolic function was assessed using two standard echocardiographic parameters:

1. FAC (%), calculated as [(end-diastolic area—end-systolic area)/end-diastolic area] × 100, reflecting RV contractile performance in the apical four-chamber view.
2. TAPSE (mm) was measured in the apical four-chamber view using M-mode echocardiography to quantify the longitudinal systolic excursion of the lateral tricuspid annulus.

Echocardiographic measurements were retrospectively abstracted from preoperative studies and interpreted by board-certified cardiologists who were blinded to patients’ clinical information and surgical status.

### Statistical analysis

Continuous variables were assessed for normality using a Shapiro–Wilk test. Normally distributed data are presented as means with standard deviations (SD), whereas non-normally distributed data are expressed as medians with interquartile ranges (IQR). Categorical variables are summarized as frequencies and percentages. Descriptive statistics included the number of observations, mean, SD, and median, minimum, maximum, and IQR, as appropriate. Comparisons of RV function parameters (FAC and TAPSE) between age groups (≤13 vs. >13 years) within each HI severity category were performed using independent samples t-tests or a Mann–Whitney *U* test, depending on data distribution. Bonferroni correction was applied for multiple comparisons to control for type I errors. Correlation analysis assessed linear associations among HI, age, FAC, and TAPSE using Pearson's correlation coefficient (r) after normality checks; when assumptions were not met, Spearman's *ρ* was examined in sensitivity analyses. ROC curve analysis was performed to determine the optimal age cutoff for predicting reduced RV systolic function (predefined as FAC < 35%), selecting the threshold that maximized Youden's J; we report area under the curve (AUC) with 95% confidence intervals (CI). To identify independent predictors of FAC and TAPSE, multiple linear regression analysis was conducted, adjusting for age, HI, body mass index (BMI), body surface area (BSA), and RV ejection fraction (RVEF); β (beta) denotes the unstandardized regression coefficient, and model assumptions (linearity, normality of residuals, homoscedasticity, and multicollinearity) were verified. All analyses were performed using IBM SPSS Statistics (version 26.0; IBM Corp., Armonk, NY, USA), and a two-tailed *p*-value < 0.05 was considered statistically significant.

## Results

**Figure 2:**
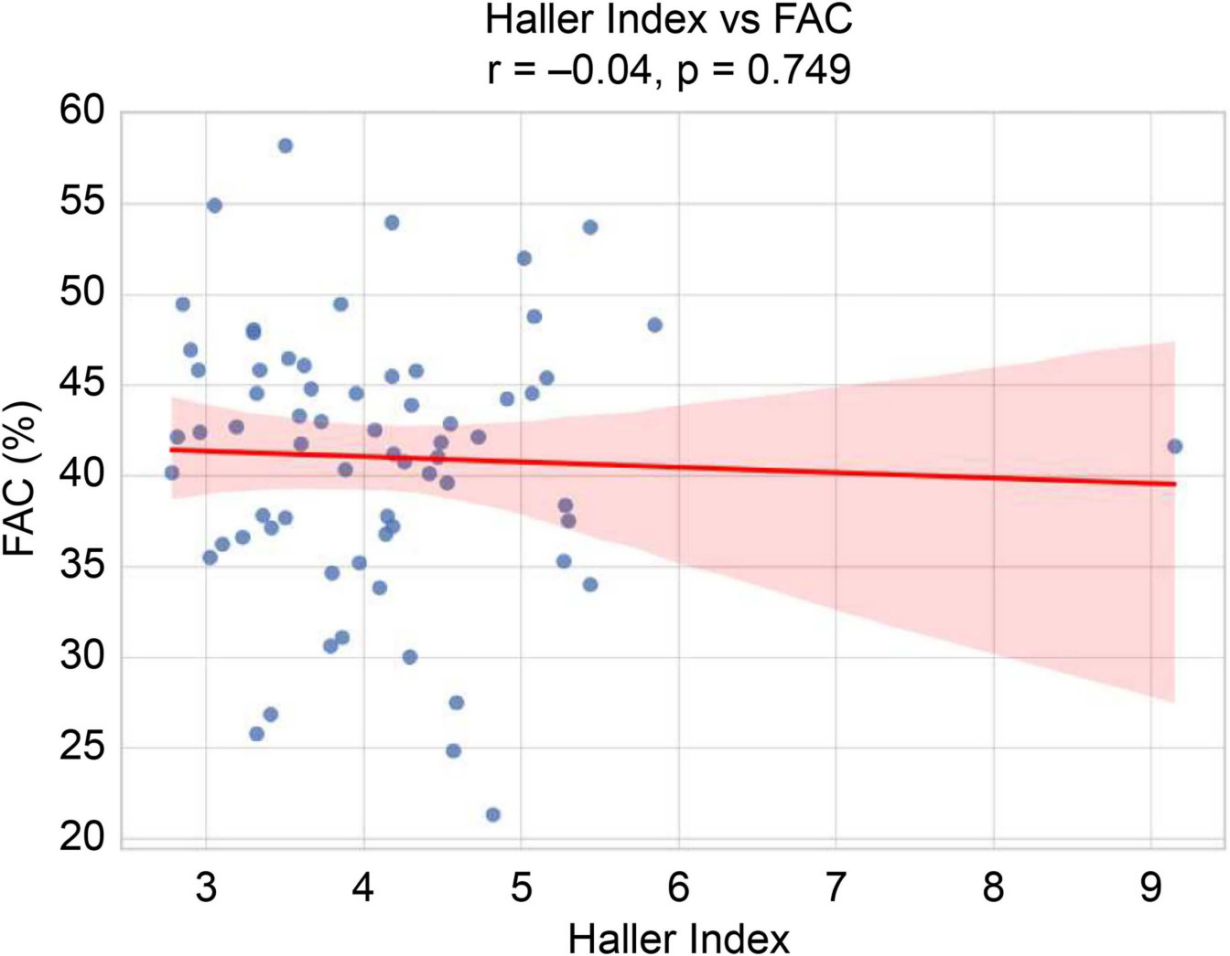
Scatter plot of haller index vs. FAC. FAC, fractional area change.

**Figure 3:**
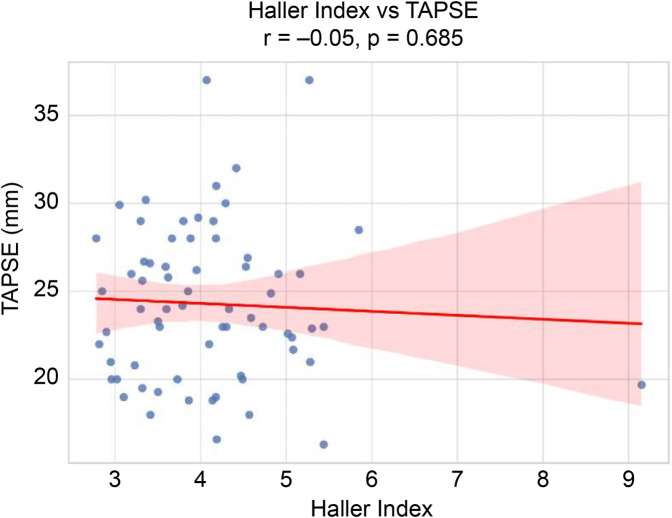
Scatter plot of haller index vs. TAPSE. TAPSE, tricuspid annular plane systolic excursion.

**Figure 4:**
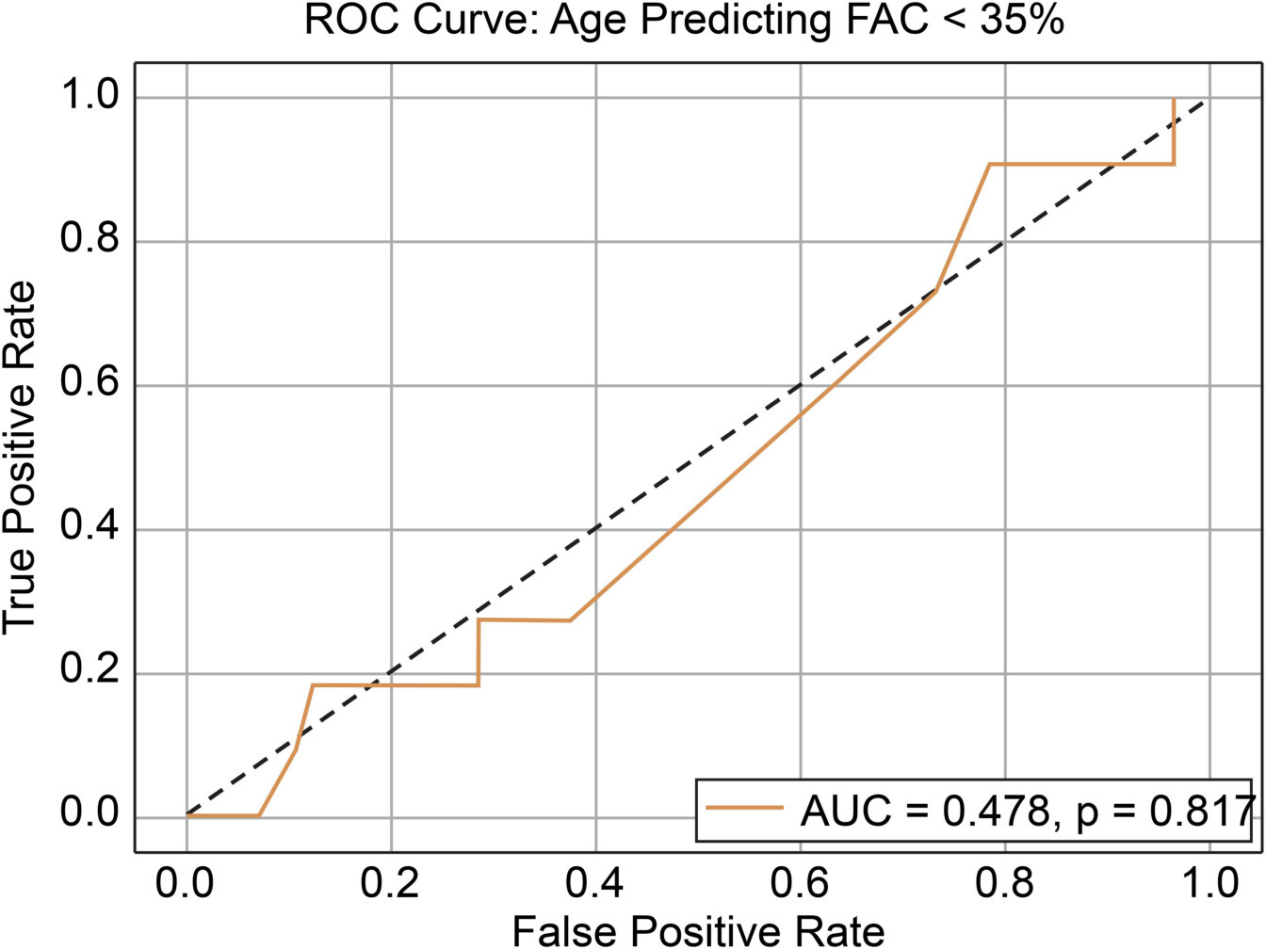
ROC curve of age predicting FAC < 35%. ROC, receiver operating characteristic; FAC, fractional area change; AUC, area under the curve.

The final study cohort comprised 67 patients (52 males and 15 females), with a mean age of 15.85 ± 4.96 (range, 3–28) years. The mean height, body weight, BMI, BSA, and HI were 165.62 cm, 50.25 kg, 18.06 kg/m^2^, 1.53 m^2^, and 4.09 ± 0.99 (range, 2.78–9.15), respectively. Based on morphological classification, 31 patients (46.3%) exhibited symmetric deformities, whereas 36 patients (53.7%) had asymmetric chest wall configurations. Preoperative echocardiographic assessment demonstrated RV systolic function, with a mean FAC of 41.03 ± 7.35% and mean TAPSE of 24.29 ± 4.41 mm. Overall, 11 of 67 patients (16.4%) met the definition of reduced RV systolic function (FAC < 35%). The baseline demographic and clinical characteristics are summarized in Table 1. Correlation analysis (Pearson's r) revealed no significant association between HI and FAC (*r* = –0.04, *p* = 0.749) (Figure 2) or between HI and TAPSE (*r* = –0.05, *p* = 0.685) (Figure 3). Similarly, age was not significantly correlated with FAC (*r* = 0.09, *p* = 0.455) or TAPSE (*r* = 0.14, *p* = 0.245) (Table 2). ROC curve analysis was performed to assess the ability of age to predict reduced RV systolic function (FAC < 35%). The area under the curve was 0.478, indicating a poor discrimination, and the optimal cutoff age—selected by maximizing Youden's J—was 13 years (Figure 4). Patients were subsequently stratified by HI severity and subdivided by age (≤13 vs. >13 years). Within each HI category, there were no statistically significant differences in FAC or TAPSE between age groups (Table 2). In the multivariable linear regression analysis adjusting for age, HI, BMI, BSA, and RVEF, only RVEF was independently associated with FAC (*β* = 0.667, *p* < 0.001) (Table 3). Other variables including age, HI, BMI, and BSA were not significantly associated with either FAC or TAPSE.

**Table 1:** Baseline patient characteristics (*n* = 67).

Variables	Value
Age	15.85 (±4.96, 3.0–28.0)
HI	4.09 (±0.99, 2.78–9.15)
FAC	41.03 (±7.35, 21.31–58.22)
TAPSE	24.29 (±4.41, 16.3–37.0)
BMI	18.06 (±3.05, 12.9–32.35)
BSA	1.53 (±0.25, 0.6–1.99)
TR Vmax	2.01 (±0.73, 0.0–2.67)

Data expressed as mean (±standard deviation, range).

HI, haller index; FAC, fractional area change; TAPSE, tricuspid annular plane systolic excursion; BMI, body mass index; BSA, body surface area; TR Vmax, tricuspid regurgitation velocity.

**Table 2:** Comparison of right ventricular function indices by age group within each haller index severity category.

HI severity group	*n* (age ≤13)	FAC (%) (age ≤13)	TAPSE (mm) (age ≤13)	*n* (age >13)	FAC (%) (age >13)	TAPSE (mm) (age >13)	*p*-value (FAC)	*p*-value (TAPSE)
Mild (<3.2)	5	40.7 ± 2.7	23.0 ± 3.9	5	46.5 ± 7.1	23.7 ± 3.9	0.146	0.778
Moderate (3.2–3.4)	1	44.5 ± —	19.5 ± —	6	40.3 ± 8.7	26.0 ± 3.4	—	—
Severe (≥3.5)	12	38.4 ± 7.4	23.9 ± 3.6	38	41.2 ± 7.5	24.8 ± 5.0	0.266	0.645

RV, right ventricle; HI, haller index; FAC, fractional area change; TAPSE, tricuspid annular plane systolic excursion.

Statistical comparison was not performed for the moderate HI group due to insufficient sample size of the ≤13 age subgroup (*n* = 1). — indicates statistics not computed (*n* = 1). Values are mean ± SD (FAC in %, TAPSE in mm). Reduced RV systolic function was predefined as FAC < 35%. Group comparisons between age subgroups within each HI stratum used independent-samples t-tests (or Mann–Whitney *U* when assumptions were not met). *r* = Pearson's correlation coefficient; *β* = unstandardized regression coefficient.

**Table 3:** Multivariate regression analysis for predictors of fractional area change and tricuspid annular plane systolic excursion (two separate models: outcome = FAC; outcome = TAPSE).

Variable	FAC β	*p*-value	TAPSE β	*p*-value
Intercept	16.375	0.307	59.825	0.033
Age	−0.073	0.519	−0.001	0.994
HI, pre	−0.188	0.716	−0.001	0.999
HI, post	0.698	0.622	−1.05	0.666
Height	−0.089	0.48	−0.224	0.304
Weight	0.112	0.461	0.068	0.795
BSA	−0.669	0.951	16.686	0.377
BMI	−0.203	0.622	−0.511	0.473
RVEF	0.667	<0.001	−0.043	0.442
RVOT Vmax (m/s)	9.295	0.16	−1.395	0.902
RVOT meanPG (mmHg)	−1.611	0.374	2.134	0.494
RVOT VTI (cm)	−0.055	0.799	0.092	0.804
MR	−0.507	0.637	2.632	0.16
TR	0.297	0.691	0.551	0.67
LVEF	−0.012	0.915	−0.335	0.095

FAC, fractional area change; TAPSE, tricuspid annular plane systolic excursion; HI, Haller index; BMI, body mass index; BSA, body surface area; RVEF, right ventricular ejection fraction; LVEF, left ventricular ejection fraction.

β denotes the unstandardized regression coefficient. Predictors in both models were age (years), Haller index (HI), BMI (kg/m^2^), BSA (m^2^), and RVEF (%). Model assumptions (linearity, normality of residuals, homoscedasticity, multicollinearity) were verified. HI, Haller index; BMI, body mass index; BSA, body surface area; RVEF, right ventricular ejection fraction.

## Discussion

In this retrospective analysis of patients with PE, neither HI nor patient age demonstrated a statistically significant association with resting RV systolic function as assessed using FAC and TAPSE. These findings are consistent with those of previous studies that questioned the reliability of anatomical severity alone in predicting physiological impairment. Even patients with markedly elevated HI values have been reported to exhibit preserved cardiac function, with no imaging evidence of RV compression (3, 4). Although functional improvements following surgical correction, such as enhanced oxygen consumption and cardiac morphology, have been documented, these outcomes have not been consistently predicted using HI alone (15). Our study adds to this body of evidence by employing both FAC and TAPSE, which are complementary echocardiographic markers, to evaluate RV function while stratifying patients according to both HI and age. This dual stratification allowed us to explore the potential influence of both anatomical deformity and compression duration. Contrary to the hypothesis that prolonged sternal depression worsens myocardial function over time, no temporal association was observed between age and RV performance. This supports prior findings that age is not a reliable predictor of cardiac dysfunction in adolescents with PE (7). Cardiopulmonary impairment in patients with PE is often more evident during exercise than at rest. Previous studies using exercise testing and stress echocardiography have shown postoperative improvements in functional capacity despite limited changes in resting parameters (16, 17). This discrepancy highlights the limitations of resting echocardiography alone and supports the integration of stress-based assessments in functional evaluations. PE is also associated with morphogenetic variability, including connective tissue disorders such as Marfan syndrome (18), which may explain the observed heterogeneity in functional impact. These structural differences are often not fully captured by HI, which quantifies only static anterior-posterior chest compressions. Moreover, skeletal maturity and thoracic compliance rather than chronological age alone may better reflect the potential mechanical restriction of cardiac function. This may explain why BSA and BMI were not associated with FAC or TAPSE, although they were included in the regression model. We also investigated whether chest wall morphology (symmetric vs. asymmetric) affected RV function and found no significant differences across age groups within each morphological subtype (Supplementary Table S1). Similarly, a reanalysis using a binary HI cutoff of 3.25, commonly cited as the surgical threshold, did not reveal age-related functional differences within the HI-defined groups (Supplementary Table S2). Taken together, these subgroup findings reinforce the limited predictive value of static anatomical severity and age alone for assessing physiological compromise. Although HI remains the most widely used index in clinical practice, it has limitations. Studies have demonstrated a substantial overlap in HI values between symptomatic and asymptomatic individuals (19), and its correlation with functional symptoms remains weak (20). Furthermore, HI does not account for the location of maximal compression or dynamic changes across the respiratory cycle (21, 22). In contrast, alternative indices such as the Correction Index (CI) have shown greater correlations with symptom burden and cardiopulmonary compromise (19, 20). Although we did not apply CI in this study, our findings support the need for more comprehensive assessment tools. This study has several limitations. This was a single-center retrospective analysis with a modest sample size, particularly within stratified subgroups. The use of resting-state TTE alone may have limited sensitivity in detecting functional impairment, especially in mildly symptomatic or athletic patients. Advanced imaging modalities, such as stress echocardiography or cardiac MRI, may better detect subtle dysfunctions, but were not available in this cohort. Furthermore, no predefined postoperative follow-up protocol was implemented; postoperative TTE and structured assessments of exercise capacity/quality of life (e.g., cardiopulmonary exercise testing, 6-minute walk test, or validated questionnaires) were not systematically collected and were therefore unavailable for analysis, limiting inferences on postoperative changes in resting RV indices and on exercise capacity/QoL. Despite these limitations, our findings emphasize the need for a multidimensional approach to evaluate the severity of PE. Reliance on HI or age alone is unlikely to adequately reflect physiological impacts. Instead, decision making should integrate functional imaging, patient symptoms, and individualized anatomical assessments. Future prospective multicenter studies incorporating dynamic and stress-based evaluations and standardized postoperative follow-up (resting TTE and objective/subjective measures of exercise capacity and QoL) are essential to refine surgical indications and identify patients who are likely to benefit from early intervention.

## Conclusion

In this retrospective cohort study, neither PE severity (measured by HI) nor patient age was significantly associated with resting RV systolic function, as assessed by FAC and TAPSE. These findings underscore the limitations of anatomical and chronological markers alone and support the integration of functional evaluation into surgical decision-making.

## Data Availability

The original contributions presented in the study are included in the article/Supplementary Material, further inquiries can be directed to the corresponding author.
