# Supplementary material for: Resting right ventricular function in pectus excavatum: do haller index and age matter?
Source: Front Surg. 2025 Nov 6;12:1685170. doi: 10.3389/fsurg.2025.1685170 (PMC12631625; doi:10.3389/fsurg.2025.1685170)
Supplement: Supplementary file 1 [file Table1.docx]

**Supplementary Table S1.** Comparison of right ventricular function by morphological type and age group

| Type | Age | n | FAC, % (Mean ± SD) | TAPSE, mm (Mean ± SD) | p-value (FAC) | p-value (TAPSE) |
| --- | --- | --- | --- | --- | --- | --- |
| Type 1 (Symmetric) | ≤13 | 8 | 36.01 ± 8.06 | 21.95 ± 3.42 | 0.36 | 0.15 |
|  | >13 | 23 | 38.99 ± 6.27 | 24.23 ± 4.43 |  |  |
| Type 2 (Asymmetric) | ≤13 | 10 | 42.05 ± 2.72 | 24.57 ± 3.46 | 0.29 | 0.79 |
|  | >13 | 26 | 43.98 ± 8.10 | 24.96 ± 4.92 |  |  |

FAC, fractional area change; TAPSE, tricuspid annular plane systolic excursion

**Supplementary Table S2**. Comparison of right ventricular functional indices (FAC and TAPSE) by Haller index and age subgroup

| Haller Index | Age | n | FAC (%) | TAPSE (mm) | p-value (FAC) | p-value (TAPSE) |
| --- | --- | --- | --- | --- | --- | --- |
| ≤ 3.25 | ≤ 13 | 5 | 40.7 ± 2.7 | 23.0 ± 3.9 | 0.251 | 0.922 |
|  | > 13 | 6 | 44.9 ± 7.5 | 23.2 ± 3.7 |  |  |
| > 3.25 | ≤ 13 | 13 | 38.8 ± 7.3 | 23.6 ± 3.6 | 0.328 | 0.325 |
|  | > 13 | 43 | 41.2 ± 7.6 | 24.8 ± 4.8 |  |  |

Data are presented as the mean ± SD

FAC, fractional area change; TAPSE, tricuspid annular plane systolic excursion; SD, standard deviation.
